# Changes in porcine nutrient transport physiology in response to *Ascaris suum* infection

**DOI:** 10.1186/s13071-021-05029-1

**Published:** 2021-10-14

**Authors:** Sarina Koehler, Andrea Springer, Nicole Issel, Stefanie Klinger, Christina Strube, Gerhard Breves

**Affiliations:** 1 Institute for Parasitology, Centre for Infection Medicine, University of Veterinary Medicine Hannover, Hanover, Germany; 2 Institute for Physiology and Cell Biology, University of Veterinary Medicine Hannover, Hanover, Germany

**Keywords:** Ascariosis, Ascariasis, Ussing chamber, Small intestine, Electrophysiology, Glucose transport, Dipeptide transport, Alanine transport

## Abstract

**Background:**

The roundworm *Ascaris suum* is one of the parasites with the greatest economic impact on pig farming. In this context, lower weight gain is hypothesized to be due to decreased nutrient absorption. This study aims at characterizing the effects of *A. suum* infection on intestinal nutrient transport processes and potential molecular mechanisms.

**Methods:**

Three groups of six piglets each were infected orally (10,000 embryonated *A. suum* eggs) in a single dose (“single infection”). Another three groups were infected orally (1000 embryonated eggs) for 10 consecutive days (“trickle infection”). Animals were necropsied 21, 35 and 49 days post-infection (dpi). Three groups served as respective controls. The Ussing chamber technique was applied for the functional characterization of small intestinal tissues [short-circuit currents (*I*_sc_) as induced by glucose, alanine and peptides; ^3^H-glucose net flux rates; tissue conductance (*G*_t_)]. Transcription and expression levels of relevant cytokines and nutrient transporters were evaluated (qPCR/western blot).

**Results:**

Peptide- and alanine-induced changes in *I*_sc_ were significantly decreased in the jejunum and ileum of the trickle-infected group at 49 dpi and in the ileum of the single-infected group at 49 dpi. No significant differences regarding glucose transport were observed between the *Ascaris*-infected groups and the control group in Ussing chamber experiments. Transcription levels of the glucose and peptide transporters as well as of selected transcription factors (transcription of signal transducer and activator of transcription 6 [*STAT6*] and hypoxia-inducible factor 1-alpha [*Hif-1α*]) were significantly increased in response to both infection types after some periods. The transcription of interleukins 4 and 13 varied between decrease and increase regarding the respective time points, as did the protein expression of glucose transporters. The expression of the peptide transporter PepT1 was significantly decreased in the ileal single-infected group at 35 dpi. Hif-1α was significantly increased in the ileal tissue from the single-infected group at 21 dpi and in the trickle-infected group at 35 dpi. The expression levels of Na^+^/K^+^-ATPase and ASCT1 remained unaffected.

**Conclusions:**

In contrast to the current hypothesis, these results indicate that the nutrient deprivation induced by *A. suum* cannot be explained by transcriptional or expression changes alone and requires further studies.

**Graphical abstract:**

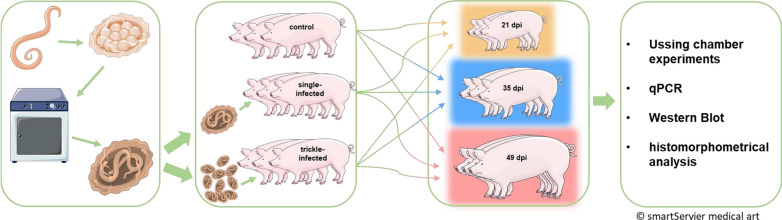

## Background

*Ascaris suum* is responsible for significant economic losses in modern pig farming [1, 2]. After oral infection with embryonated *A. suum* eggs, the larvae hatch in the stomach and small intestines, enter the vessels of the caecum and proximal colon and reach the liver [3]. Following hepatic migration, they reach the lungs, penetrate into the alveolae, are transported to the upper respiratory tract and swallowed, reaching the intestines again as from 8 dpi [4]. The worms mature in the intestines to reach the adult stage from approximately day 25–29 dpi onwards [5]. Adult *A. suum* inhabit the jejunal lumen, where each female produces up to 2 million eggs per day [6].

In Germany, up to 43% of pig livers from conventional and up to 64% from organic farming systems are condemned due to “milk spots”, i.e. hepatic scarring caused by the migrating larvae [7]. Furthermore, infected pigs show lower weight gain and feed conversion efficiency [8–11]. Lower weight gain might be explained by a dysfunction of gastrointestinal nutrient absorption induced by alterations of the intestinal ion transport [12], as well as by reduced availability of glucose or cholesterol [13]. A recent experiment confirmed a decreased glucose absorption in *A. suum*-infected pigs [14]. Chickens infected with *Ascaridia galli* also showed significantly decreased electrogenic transport of glucose and alanine [15]. These findings were associated with increased transcription of the Th2 cell-associated cytokines interleukin (IL)-4 and IL-13. IL-4 and IL-13, being upregulated during gastrointestinal nematode infections, activate signal transducer and activator of transcription 6 (STAT6), which in turn may lead to decreased sodium-linked glucose absorption [16, 17].

IL-4, IL-5 and IL-13 are secreted upon activation of Th2 cells in response to parasitic antigens, which also causes degranulation of immunoglobulin E (IgE)-sensitized mast cells [18]. One leukotriene, which is secreted by mast cells, LTD4, is known to activate the protein kinase C (PKC)-mediated phosphorylation of Raf kinase inhibitor protein, which triggers protein kinase A (PKA) to inhibit the Na-alanine cotransport in intestinal epithelial cells by decreasing the affinity of the cotransporter ASCT1 [19].

The transport of dipeptides in the small intestines as mediated by peptide transporter 1 (PepT1) depends on an inwardly directed H^+^ gradient. This gradient is established by the sodium-hydrogen antiporter 3 (NHE3). The functionality of this antiporter could be modulated by an inhibitory PKA-mediated phosphorylation of serine residues [20]. Therefore, peptide transport may be indirectly affected during *A. suum* infection.

Furthermore, phosphorylation of the sodium-glucose linked transporter 1 (SGLT1) may affect glucose transport [21–23]. Nevertheless, it is not yet clear whether phosphorylation leads to an increase or decrease in SGLT1-mediated transport. Moreover, it was shown that PKA and PKC may also modify the maximal rate of transport depending on the expressed cotransporter [24]. Also, the recruitment of glucose transporter 2 (GLUT2) to the brush border membrane [25] may modify glucose transport. Previous studies also revealed that the transcription of some genes involved in barrier function and glucose metabolism were regulated by hypoxia-inducible factor 1α (Hif-1α) [26, 27]. Hif-1α is supposedly involved in the transactivation of the *GLUT1* promoter, and therefore, an increase in Hif-1α levels might lead to increased *GLUT1* transcription [28].

The present study aimed at characterizing the mechanisms of potential malabsorption in response to *A. suum* infection in pigs. Animals were infected using a single-infection and a trickle-infection procedure. In addition to the functional characterization of intestinal nutrient transport using the Ussing chamber technique, potential mechanisms were investigated with regard to mRNA and protein expression levels.

## Methods

### Animals

Fifty-four hybrid pigs, ~5 weeks old, were acquired from the Farm for Education and Research in Ruthe, University of Veterinary Medicine Hannover, and divided into three separately housed groups. The animals received a standard pig diet ad libitum (Deuka Ferkelstarter Primo, Deutsche Tiernahrung Cremer, Düsseldorf, Germany), had free access to water and were weighed at arrival and prior to necropsy.

### Infection and sampling

Pigs of the single-infected group were each infected orally with 10,000 embryonated *A. suum* eggs, whereas pigs of the trickle-infected group received 1000 eggs/day for 10 days. Six pigs of each group and of the uninfected control group were necropsied on 21, 35 and 49 dpi. Animals were stunned with a penetrative captive bolt device and exsanguinated. The uninfected and infected pigs were necropsied alternately in the morning and afternoon. Successful infection was verified by checking livers for the presence of milk spots. Serum samples taken in weekly intervals were subjected to antibody detection by enzyme-linked immunosorbent assay (ELISA) (human anti-*Ascaris lumbricoides* IgG ELISA, Abcam (Netherlands) B.V., Amsterdam, Netherlands). Due to the close genetic relationship of *A. lumbricoides* and *A. suum*, as well as the fact that the ELISA kit utilizes protein A as secondary antibody, the test was considered suitable for use in pigs. Furthermore, macroscopically visible worms were collected during the sampling procedure for Ussing chamber experiments and molecular analyses.

To ensure the comparability of sampling, 80 cm of each intestinal segment were removed as follows: Tissue from the duodenum was taken at 80 cm distal of the pylorus, from jejunum at 6.0 m distal of the pylorus, and from ileum at 1.0 m proximal of the ileocaecal valve (the first 30 cm were discarded). Tissues for western blotting and qPCR (first 20 cm of each 80-cm segment) were rinsed with ice-cold 0.9% NaCl, stripped of the muscle layers, frozen in liquid nitrogen and stored at −80 °C until further analyses. Samples for Ussing chamber experiments (last 50 cm of each 80-cm segment) were also rinsed with ice-cold 0.9% NaCl and stored in serosal buffer (Table 1) until use.

**Table 1:** Composition [mM], osmolarity and pH of buffer solutions for Ussing chamber experiments

	Buffer I (serosal)	Buffer II (mucosal)	Buffer III (mucosal)
NaCl	113.6	113.6	113.60
KCl	5.4	5.4	5.40
HCl (1n)	0.2	0.2	0.20
MgCl_2_·6H_2_O	1.2	1.2	1.20
CaCl_2_·2H_2_O	1.2	1.2	1.20
NaHCO_3_	21.0	21.0	2.00
Na_2_HPO_4_·2H_2_O	1.5	1.5	0.37
Glucose	5.0	–	–
Mannitol	1.2	1.2	32.94
HEPES	7.0	20.0	–
Na-Gluconate	6.0	–	19.83
1n NaOH	–	6.0	–
NaH_2_PO_4_·H_2_O	–	–	1.13
Osmolarity	290 mosmol/l	292 mosmol/l	300 mosmol/l
pH	7.4	7.4	6.4

### Ussing chamber experiments

The Ussing chamber technique [29] was used to characterize the electrogenic transport of glucose, alanine and the dipeptide glycyl-l-glutamine [gly-gln] in duodenal, jejunal and ileal tissues. The tissues were incubated under voltage-clamped conditions, thus, potential differences and respective electrical currents resulting from active ion transport processes were compensated by inversely directed short-circuit currents (*I*_sc_) as a measure of net electrogenic ion transport. To control epithelial integrity, tissue conductance (*G*_t_) was measured continuously.

Tissues were opened along the mesenteric side, the serosal and the muscle layer were stripped, and the mucosa was mounted in the Ussing chambers (1.0-cm^2^ serosal area). Tissues were aerated with carbogen, and temperature was held at 37 °C. The experiment included 24 chambers per pig, of which six were used for peptide and six for alanine transport measurements (buffer I and III, Table 1), whereas 12 chambers were used for glucose transport measurements (buffer I and II, Table 1). After initial equilibration (5–10 min), potential differences were set to 0 mV, and *I*_sc_ was measured.

For the measurement of electrogenic glucose transport, glucose (5 mM, mucosal) was added after equilibration (20 min) under voltage-clamped conditions, as well as mannitol (5 mM, serosal) to prevent osmotic effects. Δ*I*_sc_ was calculated by subtracting the basal value before addition from the maximal response after addition.

Additionally, unidirectional glucose flux rates were measured by adding 5 µCi ^3^H-d-glucose (185,000 Bq) after 10 min either to the mucosal or the serosal side. Two samples (50 µl each) were taken from the radioactive side at the beginning and after 60 min of incubation. Four samples (500 µl each) were taken from the unlabelled side (15 min intervals) and mixed with 4.3 ml of scintillation liquid Rotiszint^®^ eco plus LSC-Universal cocktail (Carl Roth GmbH + Co. KG, Karlsruhe, Germany), and decays per minute (dpm) were measured (Packard 2500 TR liquid scintillation analyser, Packard BioScience Company, Meriden, CT, USA). Transepithelial active glucose transport (*J*_net_) was calculated from the difference between both unidirectional flux rates (*J*_ms_ = flux rate from the mucosal to the serosal side; *J*_sm_ = flux rate from the serosal to the mucosal side) according to standard equations [30].

The electrogenic transport of gly-gln was measured after preincubation with amastatin (0.01 mM, mucosal, 30 min). After preincubation, gly-gln (10 mM, mucosal) and mannitol (10 mM, serosal) were added. Alanine transport was measured by adding alanine (10 mM, mucosal) as well as mannitol (10 mM, serosal). At the end of each protocol, forskolin (0.01 mM, serosal) was used to test tissue viability (induction of Cl^−^ secretion).

### Quantitative real-time PCR (qPCR)

#### Design of primer–probe combinations

Nucleotide sequences of selected *Sus scrofa* genes were derived from the GenBank database, and Clone Manager (Version Professional 9, Sci Ed Software, Westminster, CO, USA) was used to align multiple sequences. Gene-specific primers and TaqMan™ MGB probes (Table 2) were designed with AlleleID software (PREMIER Biosoft, San Francisco, CA, USA). Probes (Life Technologies, Darmstadt, Germany) were labelled at the 3′ end with FAM™ or VIC^®^ to enable duplex qPCR, whereas the 5′ end was labelled with a non-fluorescent quencher.

**Table 2:** Primer pairs and TaqMan™ MGB probes for quantitative real-time PCR

Gene	Forward primer (5′–3′)	Reverse primer (5′–3′)	TaqMan™ MGB probe (5′–3′)	Amplicon length (bp)
*TBP*	CTGCCCGGTTATTTATATTTAGA	AGTCCAATCAATTGTTGAGG	VIC-ACTTACTGCTGTTGAC-NFQ	118
*PPIA*	GCAGACAAAGTTCCAAAGA	CACCCTGGCACATAAATC	6-FAM-AACTTCCGTGCTCT-NFQ	106
*GLUT1*	ATCCCATGGTTCATTGTG	CACAGTTGCTCCACATAC	VIC-AACTCTTCAGCCAG-NFQ	131
*GLUT2*	GGAAGAAGCATCAAGTGAA	GATCCCATTGATTCCAGAAA	6-FAM-CATCAGTGCTACTAGA-NFQ	127
*SGLT1*	CACTCAGTCGGATGTCTA	CCACAACTCTTAAAATAACATTCA	VIC-CACTGACATGCTGA-NFQ	133
*IL-4*	CTTCGGCACATCTACAGA	TCGTCTTTAGCCTTTCCA	6-FAM-CTCTTCTTGGCTTCA-NFQ	148
*IL-13*	CTCTGGTTGACTCTGGTC	TCTGGTTCTGGGTGATATTG	6-FAM-TTGCTCTCACCTGCTT-NFQ	127
*HIF1α*	CTGGACACAGTGTGTTTG	GCTAGTTAAGGTACACTTCATTC	VIC-TACTCATCCGTGCGAC-NFQ	149
*Stat6*	CTCAGATGCCTTCTGCTG	GTCCCTCTGATATATGCTCTC	6-FAM-TGCTATCTGCCACT-NFQ	142
*PepT1*	TCGGCTGGAATGACAATC	GGTGTAGACGATGGACAAC	VIC-TCCACTGCCATCTA-NFQ	143

#### Extraction of mRNA and cDNA synthesis

Isolation of mRNA from 30 to 40 µg mucosa of each segment was performed with the GenElute Direct mRNA Miniprep Kit (Sigma-Aldrich, Taufkirchen, Germany) with prior homogenization using the Precellys metal kit in a Precellys^®^ 24 instrument (6400 rpm, 60 s; PEQLAB Biotechnologie, Erlangen, Germany). Quality and quantity of mRNA were determined using a NanoDrop™ 1000 spectrophotometer (PEQLAB Biotechnologie), followed by overnight ethanol precipitation. For this, 100 µl sample was mixed with 5 µl 3 M sodium acetate buffer (pH 5.2), 300 µl ice-cold ethanol (95%) and 20 ng glycogen, followed by centrifugation (30 min, 16,000×*g*, 4 °C). The supernatant was removed, and 500 µl of cold ethanol (70%) was added. After centrifugation at 16,000×*g* for 10 min, the pellet was dried and resuspended in 21 µl RNAse-free water. The RNA to cDNA EcoDry™ Premix (OligodT) strips (Takara Bio Europe/Clontech, Saint-Germain-en-Laye, France) were used for cDNA synthesis of 20 μl precipitated mRNA. Quality and quantity were again determined, and cDNA was diluted 1:10 in RNAse-free water for storage at −80 °C until use.

#### Plasmid standard preparation

Plasmid standard dilution series were produced to quantify specific amplification efficiencies of the target and reference (housekeeping) genes and were included in each qPCR run. Primers were designed based on publicly available mRNA sequences (Table 3) using Primer-BLAST (NCBI). Primers were obtained from Sigma-Aldrich (Taufkirchen, Germany), and primer sequences are available on request.

**Table 3:** Accession numbers of respective gene sequences and annealing temperatures for quantitative real-time PCR

Accession number	Gene target	Annealing temperature (°C)
DQ845178	TATA box binding protein (TBP)	61
NM_214353	Peptidylprolyl isomerase A (PPIA)	60
KU672521	Glucose transporter 1 (GLUT1)	59
NM_001097417	Glucose transporter 2 (GLUT2)	60
NM_001164021	Sodium/glucose cotransporter 1 (SGLT1)	60
NM_001123124	Hypoxia inducible factor 1 subunit alpha (HIF1A)	61
NM_214123	Interleukin 4 (IL-4)	60
NM_213803	Interleukin 13 (IL-13)	59
NM_001197306	Signal transducer and activator of transcription 6 (STAT6)	61
NM_214347	Peptide transporter 1 (PepT1)	61

Partial *TBP*, *GLUT2*, *SGLT1*, *IL-13* and *STAT6* sequences were amplified in a total volume of 50 μl, including 38 μl H_2_0, 5 μl 10 × Taq buffer, 1 μl 10 mM dNTPs, 2 μl of 10 µM forward and reverse primer each and 1 μl PerfectTaq DNA polymerase (5 U/reaction, 5PRIME GmbH, Hilden, Germany). Furthermore, *PPIA*, *GLUT1*, *Hif1α*, *IL-4* and *PepT1* were amplified in a 50 μl reaction using Qiagen Multiplex Mix (Qiagen, Hilden, Germany). As template, 1 μl cDNA (200 ng/μl) derived from jejunal mucosa of a control pig was added in both protocols.

The 5 Prime Perfect Taq PCR thermocycling protocol comprised 3 min at 95 °C, 40 cycles of 95 °C for 30 s, the gene-specific annealing temperature (Table 3) for 30 s and 72 °C for 30 s, and final elongation at 72 °C for 10 min. The thermoprofile for the Qiagen Multiplex mix included 95 °C for 15 min, 40 cycles at 94 °C for 30 s, the gene-specific annealing temperature (Table 3) for 90 s, and 72 °C for 30 s, and 10 min final elongation at 72 °C.

Amplification products were ligated into TOPO™ TA vectors and transformed into One Shot™ TOP10 Chemically Competent *E. coli* (both Invitrogen™, Thermo Fisher Scientific, Schwerte, Germany). After sequencing, the plasmid-DNA was purified, linearized and dephosphorylated according to Laabs et al. [31]. Based on the absorbance at 260 nm (NanoDrop™ 1000, PEQLAB Biotechnologie), dilutions containing 10^0^ to 10^6^ copies/µl were prepared. Aliquots were stored at −20 °C for single use.

#### Quantitative real-time PCR

For each duplex qPCR reaction, 12.50 μl ABsolute™ Blue QPCR Low ROX Mix (Thermo Fisher Scientific, Schwerte, Germany), 0.15 μl of each primer (50 μM) and 0.06 μl of each probe (10 μM) were mixed. Following duplex pairs were used: PPIA/TBP, GLUT1/GLUT2, SGLT1/IL-4, Hif-1α/IL13 and PepT1/STAT6. As template, 2 μl of cDNA and 1 μl of each tenfold-diluted serial plasmid standard were used, respectively. The thermoprofile consisted of 15 min at 95 °C and 40 cycles of 95 °C for 20 s, 52 °C for 20 s and 72 °C for 30 s. The reference genes (*PPIA/TBP*) were included in each run. All reactions were run in duplicate, and each run was repeated once. The mean gene-specific amplification efficiency, correlation coefficient (*R*^2^), slope value and *y*-intercept were analysed (Table 4). Normalization of average baseline-corrected reporter signals of duplicates and technical replicates, correction of run-specific amplification efficiencies, inter-run calibration and normalization against reference genes were performed with qBase + software (Version 3.2, Biogazelle, Ghent, Belgium).

**Table 4:** Quantitative real-time PCR parameters

Gene	Amplification efficiency (%)	*R*^2^	Slope	*y*-Intercept
TBP	104.2	0.998	39.14	−3.225
PPIA	98.6	0.996	40.64	−3.355
GLUT1	97.3	0.998	40.65	−3.389
GLUT2	96.2	0.998	39.89	−3.416
SGLT1	100.8	0.993	39.48	−3.304
IL-4	102.9	0.999	40.10	−3.255
IL-13	95.7	0.998	41.21	−3.429
HIF1α	101.0	0.998	39.18	−3.298
Stat6	113.5	0.988	39.70	−3.036
PepT1	92.9	0.997	39.80	−3.506

The stability of the reference genes was controlled comparing single-infected, trickle-infected and uninfected pigs at 21, 35 and 49 dpi in each intestinal segment for each gene combination. The total average *M*-value, which represents the stability of PPIA and TBP, was 0.810, with a coefficient of variation (CV) of 0.287. As the average *M*-value remained < 1.0, the reference genes were considered stable for the normalization of the eight target genes during qPCR analyses.

### Western blot analysis

#### Preparation of cytosol, crude and apical membranes

Western blot analysis was performed for ASCT1, pSGLT1, SGLT1 and PepT1, which are located in the apical membrane, GLUT2 and Na^+^/K^+^-ATPase, located in the basolateral membrane, and cytosolic Hif-1α. To enrich apical membrane proteins and crude membranes (for the detection of the basolateral proteins), 1.5 g of tissue was thawed in 13.5 ml homogenization buffer (2 mM TRIS base, 50 mM mannitol, pH 7.1) on ice. The tissue was homogenized with a Potter tissue grinder (OMNILAB-LABORZENTRUM GmbH & Co. KG, Bremen, Germany) and centrifuged (2000×*g*, 4 °C, 15 min). The supernatant was transferred into a beaker for precipitation by dropwise addition of calcium chloride (final concentration 100 mM). Crude membranes were enriched via centrifugation (60 min, 25,830×*g*, 4 °C) of 2 ml of the homogenate. For the enrichment of the apical membrane, 7.5 ml of the homogenate was centrifuged (30 min, 2000×*g*, 4 °C), and 4 ml of the resulting supernatant was again centrifuged at 25,830×*g* (4 °C, 60 min). The pellets were resuspended (10 mM TRIS base, 150 mM NaCl, pH 7.4, phosphatase inhibitors [1:100], protease inhibitors [1:50] (protease inhibitor cocktail [P8340], phosphatase inhibitor cocktail [P5726], Sigma-Aldrich Chemie GmbH, Munich, Germany)), aliquoted and stored at −20 °C.

For cytosol preparation, 250 µl phenylmethanesulfonyl fluoride (Sigma-Aldrich Chemie GmbH, Munich, Germany) was added to 50 ml cytosol buffer (150 mM NaCl, 1% Nonidet™ P 40 [Sigma-Aldrich Chemie GmbH, Munich, Germany], 50 mM TRIS base, 5 mM EDTA). Tissue (0.2 g) was mixed with 1.85 ml of this buffer and thawed on ice. After homogenization with the Potter grinder and centrifugation (17,500×*g*, 4 °C, 30 min), the aliquoted supernatant was stored at −20 °C. Protein content was measured using the Bradford method (SERVA Electrophoresis GmbH, Heidelberg, Germany).

#### Western blotting

The samples were dissolved under reducing conditions (Table 5) in Tris–glycine buffer (125 mM TRIS HCl, 3% sodium dodecyl sulfate [SDS], 10% glycerol, 0.1 M DTT). During electrophoresis (8.5% SDS–polyacrylamide gel), Tris–glycine buffer (250 mM TRIS base, 1.92 M glycine, 1% SDS) and the 26,616 PageRuler™ (Thermo Fisher Scientific, Waltham, MA, USA) were used. Proteins were electroblotted on nitrocellulose membranes (Amersham, Freiburg, Germany). Membranes were blocked in 5% milk (5 g milk powder, Tris-buffered saline with 1% Tween 20 [TBST], 60 min, RT) and incubated overnight with the primary antibody (see Table 5) at 4 °C. For Hif-1α, all steps including TBST were performed using PBST.

**Table 5:** Denaturation and incubation conditions used in western blot experiments

Protein	Prep	Denaturation	µg/lane	Primary antibody	Secondary antibody
SGLT1^a^	AM	40 °C, 15 min	10 µg	1:2000	1:10,000^h^
pSGLT1^b^	AM	40 °C, 15 min	10 µg	1:200	1:10,000^i^
ASCT1^c^	AM	95 °C, 7 min	10 µg	1:1,000^ l^	1:2000^j^
PepT1^d^	AM	95 °C, 7 min	10 µg	1:500^ l^	1:2000^k^
GLUT2^e^	CM	70 °C, 20 min	10 µg	1:2000	1:20,000^h^^,m^
Na^+^/K^+^-ATPase^f^	CM	70 °C, 20 min	10 µg	1:10,000	1:20,000^k^
Hif-1α^g^	CY	95 °C, 5 min	10 µg	1:200^n^	1:2000^i^

*AM* apical membrane, *CM* crude membrane, *CY* cytosol

^a^Rabbit-anti-SGLT1 (ab14686, Abcam, Cambridge, UK)

^b^Custom-made (Perbio Science, Bonn, Germany; epitope: KIRKRApSEKELMI)

^c^Rabbit-anti-ASCT1 (#ANT-081, alomone labs, Jerusalem, Israel)

^d^Mouse-anti-PEPT1 (373,742, Santa Cruz, Dallas, TX, USA)

^e^Rabbit-anti-GLUT2 (ABIN310208, Antibodies-online, Aachen, Germany)

^f^Mouse-anti-Na^+^/K^+^-ATPase (ALX-804–082, Enzo Life Sciences, Farmingdale, NY, USA)

^g^Rabbit-anti-Hif-1α (#10,006,421, Cayman chemical, Ann Arbor, MI, USA)

^h^Goat-anti-rabbit HRP (A9169, Sigma-Aldrich, St. Louis, MO, USA)

^i^Goat-anti-rabbit HRP (#7074, Cell Signaling Technology, Danvers, MA, USA)

^j^m-IgGκ BP (516,102, Santa Cruz, Dallas, TX, USA)

^k^Goat-anti-mouse (A2304, Sigma-Aldrich, St. Louis, MO, USA)

^l^Diluted in BSA (5%) instead of 5% milk/TBST

^m^Diluted in 2.5% milk/TBST

^n^Diluted in 2.5% milk/PBS

Membranes were washed (3 × 10 min, TBST) and the secondary antibodies were applied for 90 min at RT. After washing (3 × 10 min, TBST), secondary antibodies were detected by use of SuperSignal^®^ West Dura Extended Duration Substrate for ASCT1, Na^+^/K^+^-ATPase, PepT1 and SGLT1. SuperSignal^®^ West Femto Maximum Sensitivity Substrate (both Thermo Fisher Scientific) was used for GLUT2, pSGLT1 and Hif-1α. Signals were detected with the ChemiDoc™ MP imaging system (Bio-Rad Laboratories, Feldkirchen, Germany). Band intensities were analysed using Image Lab Software (Bio Rad, v. 5.2.1).

For the detection of pSGLT1, the membranes incubated with the pSGLT1 antibody were stripped after the first antibody detection. For normalization, membranes were stained with Indian ink (royal blue ink combined with 2% glacial acetic acid, 30 min) and destained with distilled water.

Following molecular masses were observed: SGLT1—90 kDa, pSGLT1—95 kDa, GLUT2—58 kDa, Na^+^/K^+^-ATPase—110 kDa, PepT1—79 kDa, Hif-1α—130 kDa. Regarding ASCT1, two bands were detected, at 65 kDa and at 40 kDa. Both bands also disappeared after the addition of a specific antigenic peptide. Due to recommendations of the antibody supplier Alomone Labs™ (Jerusalem, Israel) and according to the literature [32], the 65 kDa band was chosen for evaluation.

### Histomorphometric analysis

For histomorphometric analysis of jejunal and ileal samples from 49 dpi, tissue was rinsed with ice-cold 0.9% saline and stored in 4% formalin for 2 days. After transfer to 70% ethanol, samples were cut to ~ 15–20 × 1–2 mm. The tissue was embedded in Surgipath Paraplast (Leica Biosystems Nussloch GmbH, Nussloch, Germany), and 1–2-µm slices were prepared using an HM 325 microtome (Microm GmbH, Neuss, Germany). Haematoxylin and eosin staining was carried out automatically (Leica CV 5030, Leica Biosystems Nussloch GmbH) after deparaffinization using xylene and rehydration in a descending alcohol series with subsequent transfer to tap water. Slides were digitalized with an Aperio CS2 scanner (Leica Biosystems Nussloch GmbH). Aperio ImageScope (version 12.4.3.5008, Leica Biosystems, Wetzlar, Germany) was used to evaluate 15 villi per animal. The total length, i.e. from the top of the villus to the bottom of the crypt, the villus length from the top of the villus to the beginning of the crypt, villus width and crypt depth were determined.

### Data analysis and statistics

Results are presented as means ± SEM. For creation of figures and statistical analysis, GraphPad Prism 8.4.0 was used. Mann–Whitney *U*-tests were performed, except for weight and weight gain, which were analysed with Student’s unpaired *t*-test. Differences were regarded as significant if *P* < 0.05.

## Results

All single- and trickle-infected pigs showed liver milk spots upon slaughter, indicating successful infection, while no milk spots were noted in the control group. Regarding antibody detection, all control pigs remained seronegative throughout the study period, while most infected pigs seroconverted between 7 and 21 dpi (22/36 infected pigs). It should be noted that three pigs seroconverted particularly late during the study period, after 35 dpi. Consequently, 4/6 single-infected and 5/6 trickle-infected pigs each were seropositive upon slaughter at 21 and 35 dpi, while all single-infected pigs and 4/6 trickle-infected pigs slaughtered at 49 dpi had seroconverted. Macroscopically visible worms were collected from the intestinal contents of 10 pigs and microscopically verified as *A. suum*.

Based on the Ussing chamber experiments, which showed no significant changes throughout the experiment in either infection type in the duodenum, only the results of the jejunum and ileum are presented.

### Weight gain

There were no significant differences in weight gain between the groups at 21 dpi and 35 dpi. At 49 dpi, there was a significantly increased weight gain in the trickle-infected group (Table 6).

**Table 6:** Summary of initial and final weight [kg] and calculated weight gain (means ± SEM)

	Control	Single infection	Trickle infection	Single infection vs control	Trickle infection vs control
21 dpi					
Initial	10.7 ± 0.5	10.9 ± 0.2	12.9 ± 0.8	*t* = 0.33, *P* = 0.745	*t* = 2.29, ***P* = 0.045**
Final	22.0 ± 0.5	21.7 ± 0.7	26.6 ± 1.8	*t* = 0.33, *P* = 0.750	*t* = 2.55, ***P* = 0.029**
Gain	11.3 ± 0.8	10.8 ± 0.8	13.7 ± 1.0	*t* = 0.37, *P* = 0.707	*t* = 1.79, *P* = 0.104
35 dpi					
Initial	9.2 ± 0.2	9.7 ± 0.1	11.8 ± 0.3	*t* = 2.57, ***P* = 0.028**	*t* = 8.54, ***P* < 0.001**
Final	27.7 ± 1.3	29.4 ± 0.9	31.8 ± 0.7	*t* = 1.08, *P* = 0.304	*t* = 2.85, ***P* = 0.017**
Gain	18.4 ± 1.3	19.7 ± 1.0	20.1 ± 0.6	*t* = 0.75, *P* = 0.469	*t* = 1.13, *P* = 0.285
49 dpi					
Initial	8.2 ± 0.1	8.1 ± 0.3	10.3 ± 0.2	*t* = 0.19, *P* = 0.857	*t* = 11.32, ***P* < 0.001**
Final	32.8 ± 1.6	33.3 ± 1.7	40.7 ± 1.5	*t* = 0.18, *P* = 0.863	*t* = 3.57, ***P* = 0.005**
Gain	24.6 ± 1.7	25.1 ± 1.9	30.3 ± 1.5	*t* = 0.18, *P* = 0.859	*t* = 2.52, ***P* = 0.031**

Significant *P*-values in Student’s unpaired *t*-test are printed in bold

### Ussing chamber experiments

**Fig. 1:**
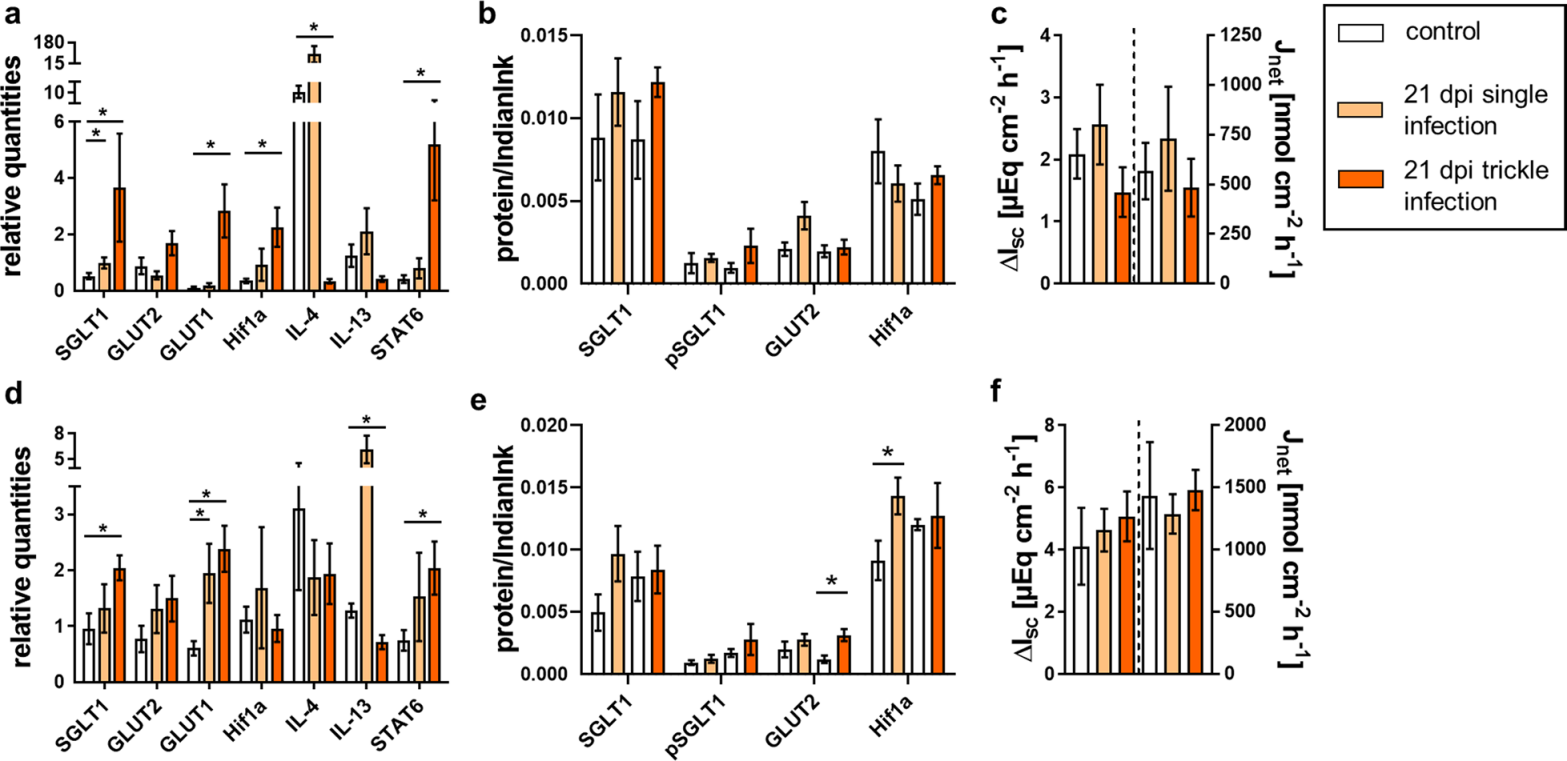
Glucose transport-associated results of single- and trickle-infected pigs (21 dpi) compared to the control group: relative quantities from jejunal (**a**) and ileal qPCR analyses (**d**), relative protein content for immunoblot analyses of jejunal (**b**) and ileal mucosa (**e**) and results of Ussing chamber measurements of jejunal (**c**) and ileal mucosa (**f**). Results of Ussing chamber experiments show the short-circuit current (∆*I*_sc_) in response to mucosal addition of 5 mM glucose (left *y* axis) and the calculated 3H-glucose net flux rate after the addition of 5 µCi 3H-d-glucose (*J*_net_, right *y* axis). Data are shown as means ± SEM, and significant differences are indicated with an asterisk (Mann–Whitney *U*-test, *P* < 0.05)

**Fig. 2:**
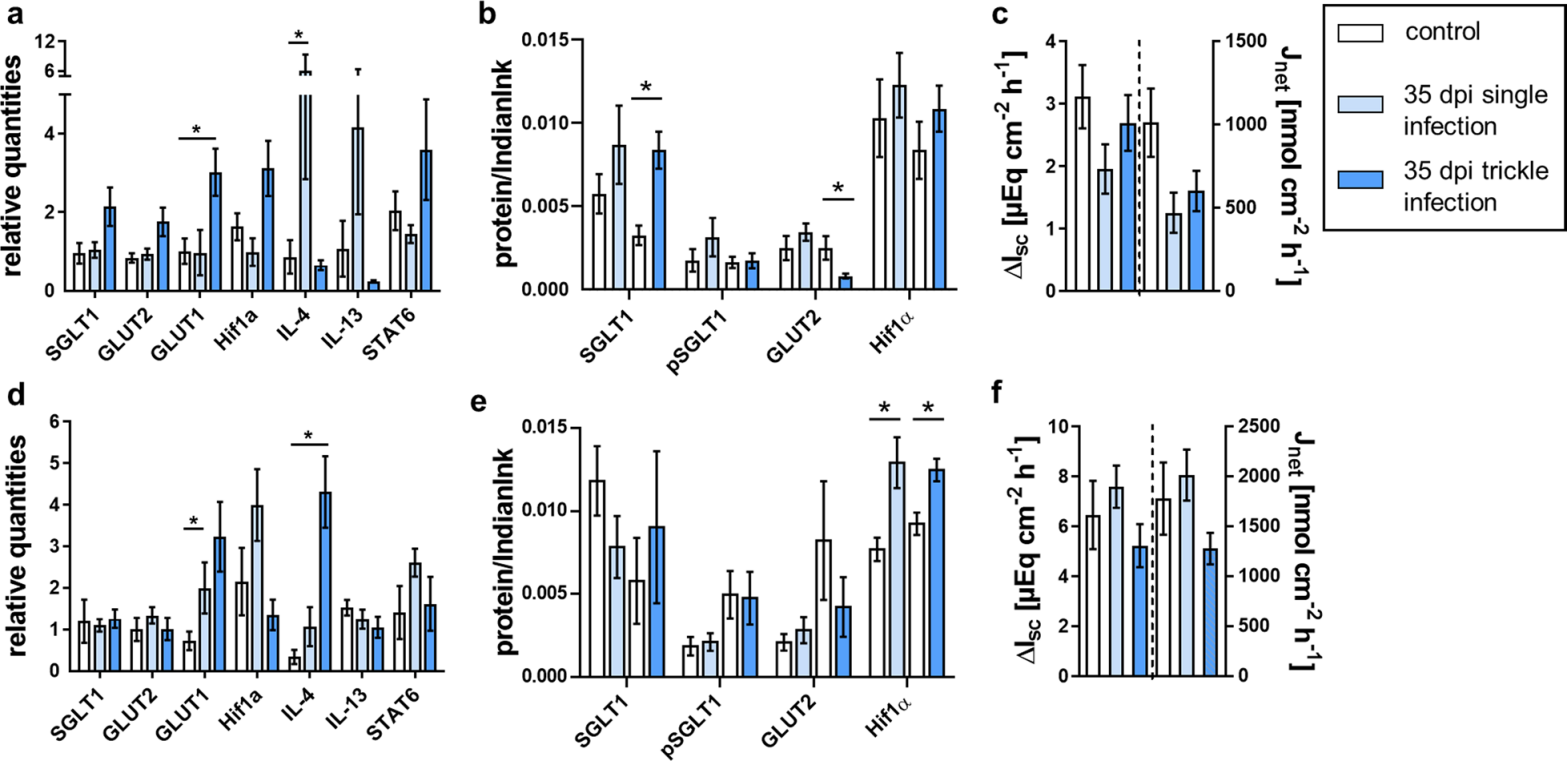
Glucose transport-associated results of single- and trickle-infected pigs (35 dpi) compared to the control group: relative quantities from jejunal (**a**) and ileal qPCR analyses (**d**), relative protein content for immunoblot analyses of jejunal (**b**) and ileal mucosa (**e**) and results of Ussing chamber measurements of jejunal (**c**) and ileal mucosa (**f**). Results of Ussing chamber experiments show the short-circuit current (∆*I*_sc_) in response to mucosal addition of 5 mM glucose (left *y* axis) and the calculated 3H-glucose net flux rate after the addition of 5 µCi 3H-d-glucose (*J*_net_, right *y* axis). Data are shown as means ± SEM, and significant differences are indicated with an asterisk (Mann–Whitney *U*-test,* P* < 0.05)

**Fig. 3:**
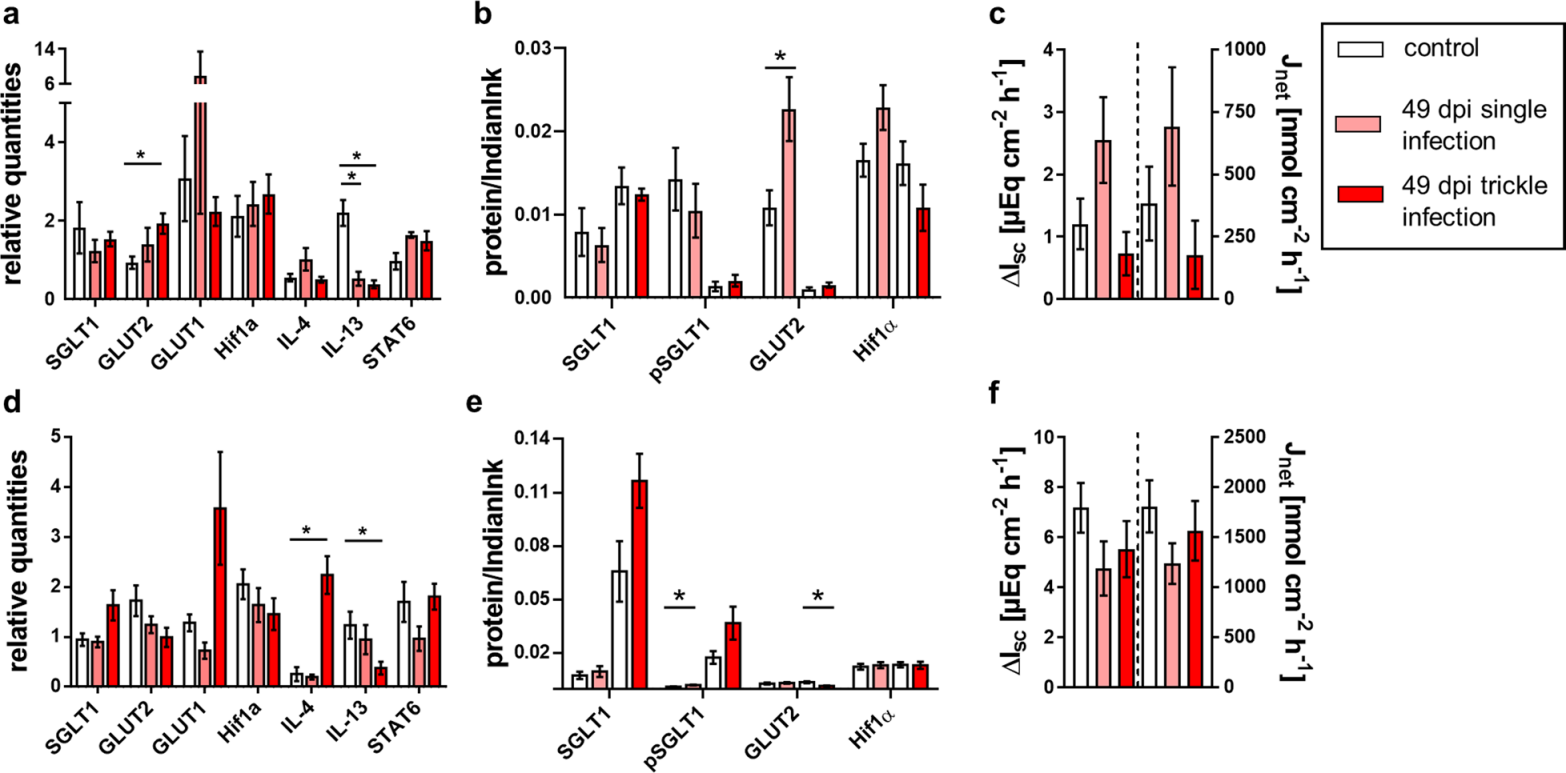
Glucose transport-associated results of single- and trickle-infected pigs (49 dpi) compared to the control group: relative quantities from jejunal (**a**) and ileal qPCR analyses (**d**), relative protein content for immunoblot analyses of jejunal (**b**) and ileal mucosa (**e**) and results of Ussing chamber measurements of jejunal (**c**) and ileal mucosa (**f**). Results of Ussing chamber experiments show the short-circuit current (∆*I*_sc_) in response to mucosal addition of 5 mM glucose (left *y* axis) and the calculated 3H-glucose net flux rate after the addition of 5 µCi 3H-d-glucose (*J*_net_, right *y* axis). Data are shown as means ± SEM, and significant differences are indicated with an asterisk (Mann–Whitney *U*-test,* P* < 0.05)

No significant differences in electrogenic glucose transport or glucose flux rates were observed between the infected animals and the control group throughout the study (Tables 7, 8, 9; Figs. 1c, f, 2c, f, 3c, f).

**Table 7:** Summary of statistical comparisons (Mann–Whitney *U*-tests) regarding mRNA, protein expression and functional data on 21 dpi

	Single infection vs control			Trickle infection vs control		
	mRNA	Protein	Ussing chamber	mRNA	Protein	Ussing chamber
21 dpi jejunum						
SGLT1	*U* = 4, ***P* = 0.026**	*U* = 13, *P* = 0.485	ΔI_sc glucose_: *U* = 11, *P* = 0.310	*U* = 4, ***P* = 0.026**	*U* = 11, *P* = 0.310	Δ*I*_sc glucose_: *U* = 11, *P* = 0.310
pSGLT1	nd	*U* = 9, *P* = 0.180	J_net glucose_: *U* = 16, *P* = 0.818	nd	*U* = 10, *P* = 0.240	*J*_net glucose_: *U* = 16, *P* = 0.818
GLUT1	*U* = 14, *P* = 0.589	nd		*U* = 0, ***P* = 0.002**	nd	
GLUT2	*U* = 14, *P* = 0.589	*U* = 9, *P* = 0.180		*U* = 9, *P* = 0.180	*U* = 16, *P* = 0.818	
PepT1	*U* = 13, *P* = 0.485	*U* = 7, *P* = 0.093	ΔI_sc gly-gln_: *U* = 15, *P* = 0.699	*U* = 1, ***P* = 0.004**	*U* = 11, *P* = 0.310	Δ*I*_sc gly-gln_: *U* = 14, *P* = 0.589
ASCT1	nd	*U* = 9, *P* = 0.180	ΔI_sc alanine_: *U* = 11, *P* = 0.310	nd	*U* = 15, *P* = 0.699	Δ*I*_sc alanine_: *U* = 15, *P* = 0.699
Na^+^/K^+^-A	nd	*U* = 6, *P* = 0.065		nd	*U* = 9, *P* = 0.180	
Hif1α	*U* = 15, *P* = 0.699	*U* = 13, *P* = 0.485		*U* = 0, ***P* = 0.002**	*U* = 12, *P* = 0.394	
IL-4	*U* = 12, *P* = 0.394	nd		*U* = 0, ***P* = 0.002**	nd	
IL-13	*U* = 10, *P* = 0.240	nd		*U* = 7, *P* = 0.093	nd	
STAT6	*U* = 16, *P* = 0.818	nd		*U* = 0, ***P* = 0.002**	nd	
21 dpi ileum						
SGLT1	*U* = 15, *P* = 0.699	*U* = 10, *P* = 0.240	ΔI_sc glucose_: *U* = 17, *P* = 0.853	*U* = 4, **P = 0.026**	*U* = 16, *P* = 0.818	Δ*I*_sc glucose_: *U* = 17, *P* = 0.937
pSGLT1	nd	*U* = 14, *P* = 0.589	J_net glucose_: *U* = 16, *P* = 0.818	nd	*U* = 17, *P* = 0.937	J_net glucose_: *U* = 18, *P* > 0.999
GLUT1	*U* = 5, *P* = 0.041	nd		*U* = 0, ***P* = 0.002**	nd	
GLUT2	*U* = 12, *P* = 0.393	*U* = 10, *P* = 0.240		*U* = 9, *P* = 0.180	*U* = 3, *P* = 0.015	
PepT1	*U* = 15, *P* = 0.699	*U* = 17, *P* = 0.937	ΔI_sc gly-gln_: *U* = 11, *P* = 0.310	*U* = 4, ***P* = 0.026**	*U* = 17, *P* = 0.937	Δ*I*_sc gly-gln_: *U* = 6, *P* = 0.065
ASCT1	nd	*U* = 7, *P* = 0.093	ΔI_sc alanine_: *U* = 15, *P* = 0.699	nd	*U* = 15, *P* = 0.699	Δ*I*_sc alanine_: *U* = 11, *P* = 0.310
Na^+^/K^+^-A	nd	*U* = 16, *P* = 0.818		nd	*U* = 12, *P* = 0.394	
Hif1α	*U* = 11, *P* = 0.310	*U* = 5, ***P* = 0.041**		*U* = 15, *P* = 0.699	*U* = 11, *P* = 0.310	
IL-4	*U* = 14, *P* = 0.589	nd		*U* = 17, *P* = 0.937	nd	
IL-13	*U* = 6, *P* = 0.065	nd		*U* = 4, ***P* = 0.026**	nd	
STAT6	*U* = 16, *P* = 0.818	nd		*U* = 4, ***P* = 0.026**	nd	

*nd.* not determined, *Na*^*+*^*/K*^*+*^*-A* Na^+^/K^+^-ATPase

Significant *P*-values are printed in bold

**Table 8:** Summary of statistical comparisons (Mann–Whitney *U-*tests) regarding mRNA, protein expression and functional data on 35 dpi

	Single infection vs control			Trickle infection vs control		
	mRNA	Protein	Ussing chamber	mRNA	Protein	Ussing chamber
35 dpi jejunum						
SGLT1	*U* = 15, *P* = 0.699	*U* = 56, *P* = 0.392	Δ*I*_sc glucose_: *U* = 8, *P* = 0.132	*U* = 7, *P* = 0.093	*U* = 1, ***P* = 0.004**	Δ*I*_sc glucose_: *U* = 15, *P* = 0.699
pSGLT1	nd	*U* = 12, *P* = 0.394	*J*_net glucose_: *U* = 6, *P* = 0.065	nd	*U* = 18, *P* > 0.999	*J*_net glucose_: *U* = 7, *P* = 0.093
GLUT1	*U* = 10, *P* = 0.240	nd		*U* = 3, ***P* = 0.015**	nd	
GLUT2	*U* = 16, *P* = 0.818	*U* = 11, *P* = 0.310		*U* = 6, *P* = 0.065	*U* = 3, ***P* = 0.015**	
PepT1	*U* = 9, *P* = 0.180	*U* = 17, *P* = 0.937	Δ*I*_sc gly-gln_: *U* = 16, *P* = 0.818	*U* = 11, *P* = 0.305	*U* = 6, *P* = 0.065	Δ*I*_sc gly-gln_: *U* = 10, *P* = 0.240
ASCT1	nd	*U* = 15, *P* = 0.699	Δ*I*_sc alanine_: *U* = 16, *P* = 0.818	nd	*U* = 10, *P* = 0.240	Δ*I*_sc alanine_: *U* = 10, *P* = 0.240
Na^+^/K^+^-A	nd	*U* = 7, *P* = 0.093		nd	*U* = 18, *P* > 0.999	
Hif1α	*U* = 9, *P* = 0.180	*U* = 14, *P* = 0.589		*U* = 7, *P* = 0.093	*U* = 9, *P* = 0.180	
IL-4	*U* = 3, ***P* = 0.015**	nd		*U* = 15, *P* = 0.699	nd	
IL-13	*U* = 6, *P* = 0.065	nd		*U* = 13, *P* = 0.485	nd	
STAT6	*U* = 6, *P* = 0.065	nd		*U* = 13, *P* = 0.485	nd	
35 dpi ileum						
SGLT1	*U* = 13, *P* = 0.485	*U* = 9, *P* = 0.180	Δ*I*_sc glucose_: *U* = 15, *P* = 0.699	*U* = 13, *P* = 0.485	*U* = 17, *P* = 0.937	Δ*I*_sc glucose_: *U* = 13, *P* = 0.485
pSGLT1	nd	*U* = 13, *P* = 0.485	*J*_net glucose_: *U* = 15, *P* = 0.699	nd	*U* = 16, *P* = 0.818	*J*_net glucose_: *U* = 12, *P* = 0.394
GLUT1	*U* = 5, ***P* = 0.041**	nd		*U* = 6, *P* = 0.065	nd	
GLUT2	*U* = 11, *P* = 0.310	*U* = 14, *P* = 0.589		*U* = 18, *P* > 0.999	*U* = 12, *P* = 0.394	
PepT1	*U* = 9, *P* = 0.180	*U* = 4, ***P* = 0.026**	Δ*I*_sc gly-gln_: *U* = 11, *P* = 0.310	*U* = 16, *P* = 0.818	*U* = 7, *P* = 0.093	Δ*I*_sc gly-gln_: *U* = 15, *P* = 0.699
ASCT1	nd	nd	Δ*I*_sc alanine_: *U* = 17, *P* = 0.937	nd	nd	Δ*I*_sc alanine_: *U* = 12, *P* = 0.394
Na^+^/K^+^-A	nd	*U* = 17, *P* = 0.937			*U* = 16, *P* = 0.818	
Hif1α	*U* = 7, *P* = 0.093	*U* = 2, ***P* = 0.009**		*U* = 15, *P* = 0.699	*U* = 3, ***P* = 0.015**	
IL-4	*U* = 8, *P* = 0.132	nd		*U* = 0, ***P* = 0.002**	nd	
IL-13	*U* = 16, *P* = 0.818	nd		*U* = 8, *P* = 0.132	nd	
STAT6	*U* = 6, *P* = 0.065	nd		*U* = 10, *P* = 0.240	nd	

*nd* not determined, *Na*^*+*^*/K*^*+*^*-A* Na^+^/K^+^-ATPase

Significant *P*-values are printed in bold

**Table 9:** Summary of statistical comparisons (Mann–Whitney *U*-tests) regarding mRNA, protein expression and functional data on 49 dpi

	Single infection vs control			Trickle infection vs control		
	mRNA	Protein	Ussing chamber	mRNA	Protein	Ussing chamber
49 dpi jejunum						
SGLT1	*U* = 16, *P* = 0.818	*U* = 12, *P* = 0.394	ΔI_sc glucose_: *U* = 7, *P* = 0.093	*U* = 18, *P* = > 0.999	*U* = 16, *P* = 0.818	ΔI_sc glucose_: *U* = 10, *P* = 0.240
pSGLT1	nd	*U* = 16, *P* = 0.818	J_net glucose_: *U* = 11, *P* = 0.310	nd	*U* = 13, *P* = 0.489	J_net glucose_: *U* = 7, *P* = 0.093
GLUT1	*U* = 17, *P* = 0.937	nd		*U* = 14, *P* = 0.588	nd	
GLUT2	*U* = 12, *P* = 0.394	*U* = 4, ***P* = 0.026**		*U* = 3, ***P* = 0.015**	*U* = 11, *P* = 0.310	
PepT1	*U* = 14, *P* = 0.589	*U* = 12, *P* = 0.394	ΔI_sc gly-gln_: *U* = 8, *P* = 0.132	*U* = 8, *P* = 0.132	*U* = 15, *P* = 0.699	ΔI_sc gly-gln_: *U* = 5, ***P* = 0.041**
ASCT1	nd	*U* = 6, *P* = 0.065	ΔI_sc alanine_: *U* = 17, *P* = 0.947	nd	*U* = 8, *P* = 0.132	ΔI_sc alanine_: *U* = 4, ***P* = 0.026**
Na^+^/K^+^-A	nd	*U* = 18, *P* > 0.999		nd	*U* = 7, *P* = 0.093	
Hif1α	*U* = 15, *P* = 0.699	*U* = 8, *P* = 0.132		*U* = 12, *P* = 0.394	*U* = 10, *P* = 0.240	
IL-4	*U* = 12, *P* = 0.394	nd		*U* = 16, *P* = 0.818	nd	
IL-13	*U* = 1, ***P* = 0.004**	nd		*U* = 0, ***P* = 0.002**	nd	
STAT6	*U* = 8, *P* = 0.132	nd		*U* = 9, *P* = 0.180	nd	
49 dpi ileum						
SGLT1	*U* = 16.5, *P* = 0.853	*U* = 14, *P* = 0.589	ΔI_sc glucose_: *U* = 8, *P* = 0.132	*U* = 8, *P* = 0.132	*U* = 6, *P* = 0.065	ΔI_sc glucose_: *U* = 16, *P* = 0.818
pSGLT1	nd	*U* = 3, ***P* = 0.015**	J_net glucose_: *U* = 7, *P* = 0.093	nd	*U* = 7, *P* = 0.093	J_net glucose_: *U* = 18, *P* > 0.999
GLUT1	*U* = 7, *P* = 0.093	nd		*U* = 6, *P* = 0.065	nd	
GLUT2	*U* = 11, *P* = 0.310	*U* = 13, *P* = 0.485		*U* = 7, *P* = 0.093	*U* = 3, *P* = *0.015*	
PepT1	*U* = 10, *P* = 0.240	*U* = 18, *P* > 0.999	ΔI_sc gly-gln_: *U* = 0, ***P* = 0.002**	*U* = 6, *P* = 0.065	*U* = 17, *P* = 0.937	ΔI_sc gly-gln_: *U* = 0, ***P* = 0.002**
ASCT1	nd	*U* = 12, *P* = 0.394	ΔI_sc alanine_: *U* = 6, *P* = 0.065	nd	*U* = 12, *P* = 0.394	ΔI_sc alanine_: *U* = 4, ***P* = 0.026**
Na^+^/K^+^-A	nd	*U* = 13, *P* = 0.485		nd	*U* = 11, *P* = 0.310	
Hif1α	*U* = 15, *P* = 0.699	*U* = 17, *P* = 0.937		*U* = 10, *P* = 0.240	*U* = 16, *P* = 0.818	
IL-4	*U* = 14, *P* = 0.589	nd		*U* = 1, ***P* = 0.004**	nd	
IL-13	*U* = 14, *P* = 0.589	nd		*U* = 2, ***P* = 0.009**	nd	
STAT6	*U* = 9, *P* = 0.180	nd		*U* = 18, *P* > 0.999	nd	

*nd* not determined, *Na*^*+*^*/K*^*+*^*-A* Na^+^/K^+^-ATPase

Significant *P*-values are printed in bold

**Fig. 4:**
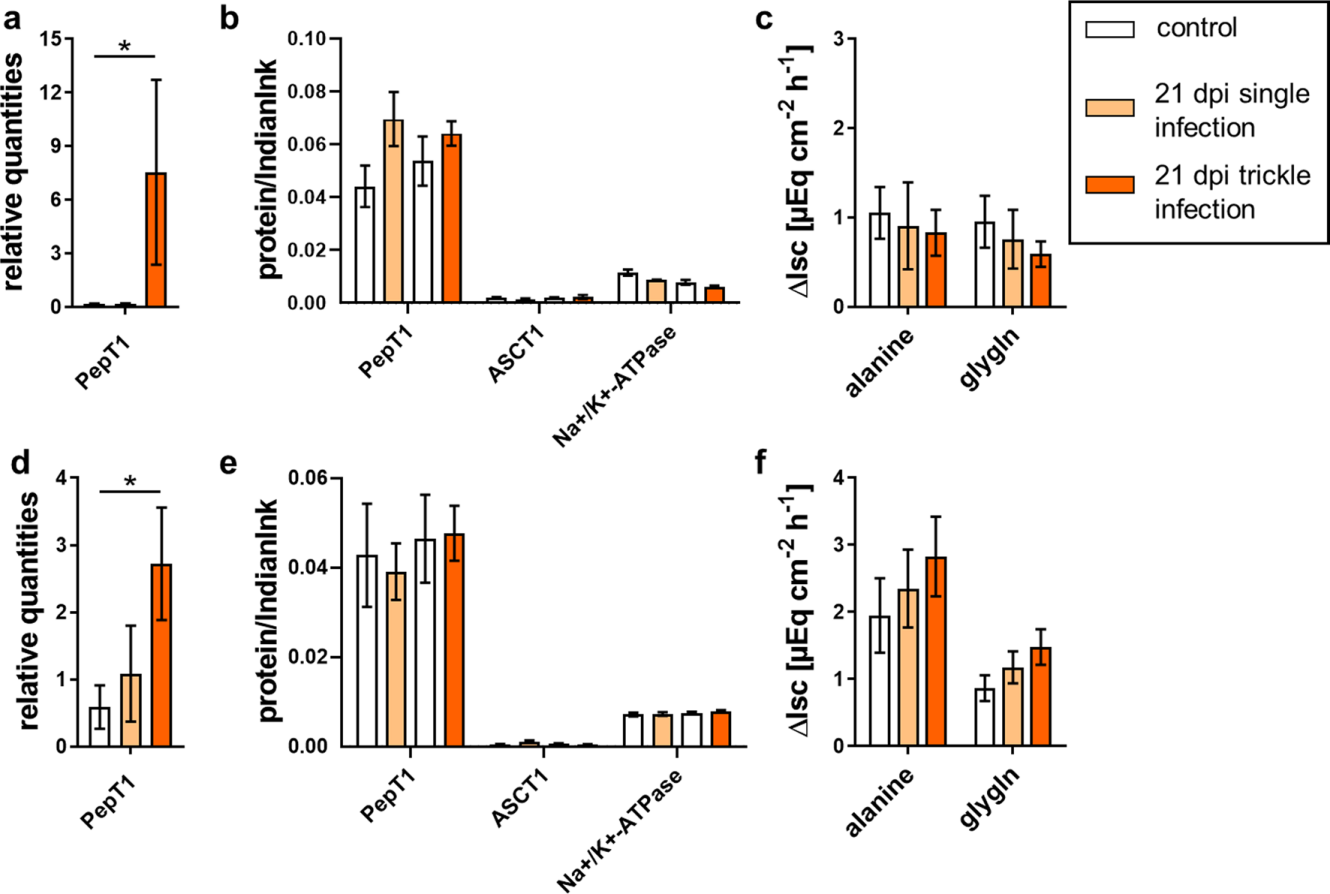
Alanine and gly-gln transport associated results of single- and trickle-infected pigs (21 dpi) compared to the control group: relative quantities from jejunal (**a**) and ileal qPCR analyses (**d**), relative protein content for immunoblot analyses of jejunal (**b**) and ileal mucosa (**e**) and results of Ussing chamber measurements of jejunal (**c**) and ileal mucosa (**f**). Results of Ussing chamber experiments show the short-circuit current (∆*I*_sc_) in response to mucosal addition of 10 mM alanine or gly-gln. Data are shown as means ± SEM, and significant differences are indicated with an asterisk (Mann–Whitney *U*-test,* P* < 0.05)

**Fig. 5:**
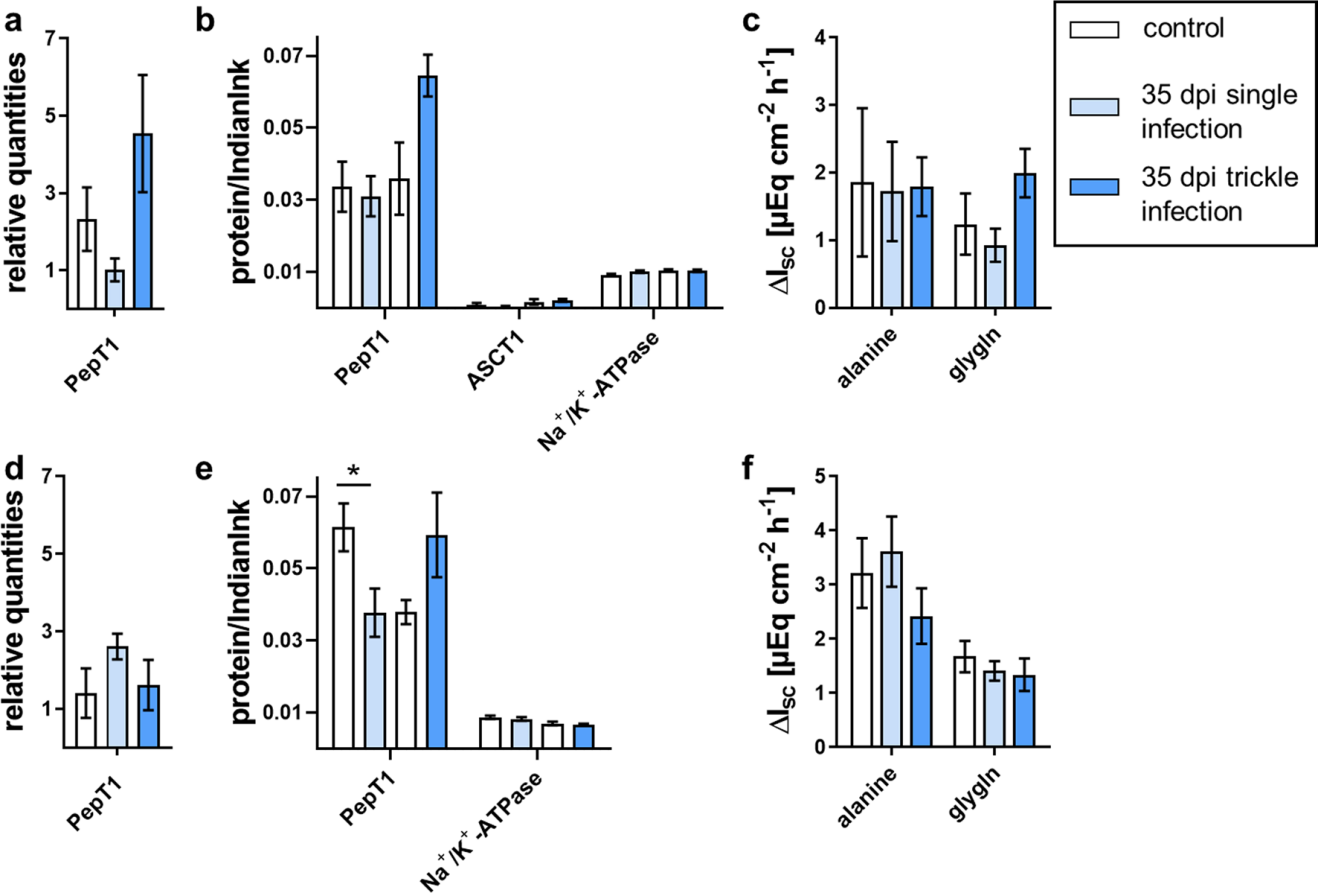
Alanine and gly-gln transport-associated results of single- and trickle-infected pigs (35 dpi) compared to the control group: relative quantities from jejunal (**a**) and ileal qPCR analyses (**d**), relative protein content for immunoblot analyses of jejunal (**b**) and ileal mucosa (**e**) and results of Ussing chamber measurements of jejunal (**c**) and ileal mucosa (**f**). Results of Ussing chamber experiments show the short-circuit current (∆*I*_sc_) in response to mucosal addition of 10 mM alanine or gly-gln. Data are shown as means ± SEM, and significant differences are indicated with an asterisk (Mann–Whitney *U*-test,* P* < 0.05)

**Fig. 6:**
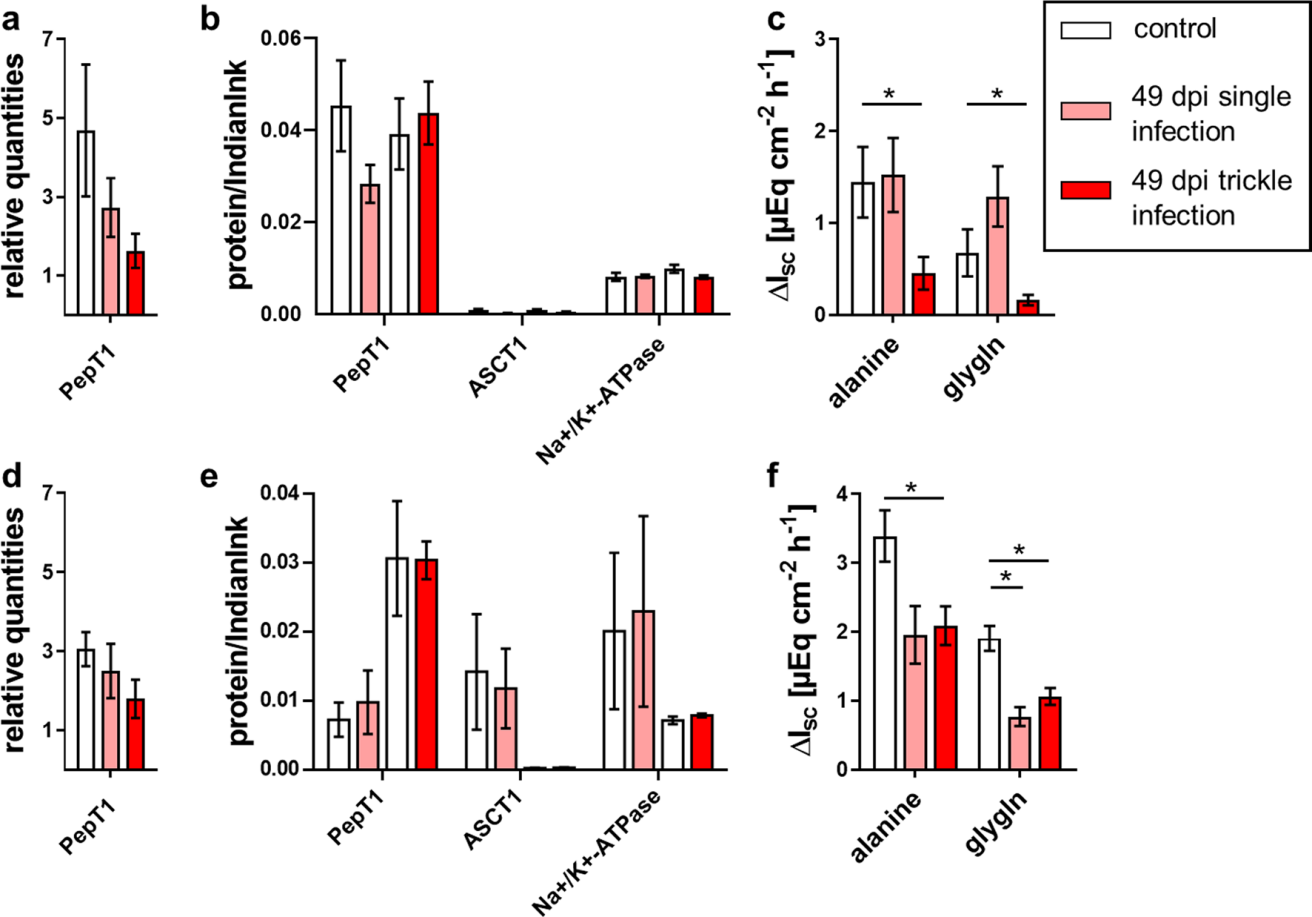
Alanine and gly-gln transport-associated results of single- and trickle-infected pigs (49 dpi) compared to the control group: relative quantities from jejunal (**a**) and ileal qPCR analyses (**d**), relative protein content for immunoblot analyses of jejunal (**b**) and ileal mucosa (**e**) and results of Ussing chamber measurements of jejunal (**c**) and ileal mucosa (**f**). Results of Ussing chamber experiments show the short-circuit current (∆*I*_sc_) in response to mucosal addition of 10 mM alanine or gly-gln. Data are shown as means ± SEM, and significant differences are indicated with an asterisk (Mann–Whitney *U*-test,* P* < 0.05)

For gly-gln, a continuous increase in the electrogenic response was noticeable along the intestinal axis of the jejunum and ileum, resulting in the highest ΔI_sc_ in the ileum. No significant differences among groups were observed at 21 and 35 dpi (Fig. 4c, f, 5c, f; Tables 7, 8). At 49 dpi, the trickle-infected group showed a significantly decreased Δ*I*_sc_ in the jejunum, while a significant decrease was observed in both infection groups in the ileum (Fig. 6c, f; Table 9). Regarding alanine transport, the electrogenic response of trickle-infected pigs showed a significant decrease in the jejunum and ileum at 49 dpi (Fig. 6c, f; Table 9).

Basal tissue conductance did not differ significantly between control and infected animals, except for significantly lower conductance at 49 dpi in the jejunum of the trickle-infected group (control group: 29.66 ± 1.57 mS/cm^2^, trickle-infected: 19.08 ± 1.83 mS/cm^2^, *U* = 14, *P* < 0.0001).

### qPCR results

At 21 dpi, a significant increase in *SGLT1* transcription was observed in the jejunum of both infection groups and in the ileum of the trickle-infected group (Fig. 1a, d; Table 7). *GLUT1* transcription was significantly increased in the jejunum of the trickle-infected group and the ileum of both infection groups at 21 dpi as well as in the jejunum of the trickle-infected and in the ileum of the single-infected group at 35 dpi (Figs. 1a, d, 2a, d, Tables 7, 8). At 49 dpi, the jejunum of the trickle-infected group showed significantly increased *GLUT2* transcription (Fig. 3a; Table 9). Only the jejunum of the single-infected group at 21 dpi showed significantly increased transcription of *Hif-1α* (Fig. 1a; Table 7).

A significant increase in *PepT1* transcription was noted in the jejunum and ileum of the trickle-infected group at 21 dpi (Fig. 4a, d; Table 7).

Transcription of *IL-4* in the jejunum was significantly decreased at 21 dpi in the trickle-infected and 35 dpi in the single-infected group (Figs. 1a, 2a; Tables 7, 8), whereas a significant increase was detected in the ileum of the trickle-infected group at 35 and 49 dpi (Figs. 2d, 3d; Tables 8, 9). For *IL-13*, a significant decrease was observed in the ileum of the trickle-infected group at 21 dpi (Fig. 1d; Table 7) and in the jejunum of both infection groups as well as in the ileum of the trickle-infection group at 49 dpi (Fig. 3a, d; Table 9). *STAT6* transcription was only affected in the trickle-infection group, with a significant increase in the jejunum and ileum at 21 dpi (Fig. 1a, d; Table 7).

### Western blot analysis

#### Expression of glucose transporters, Na^+^/K^+^-ATPase and Hif-1α

**Fig. 7:**
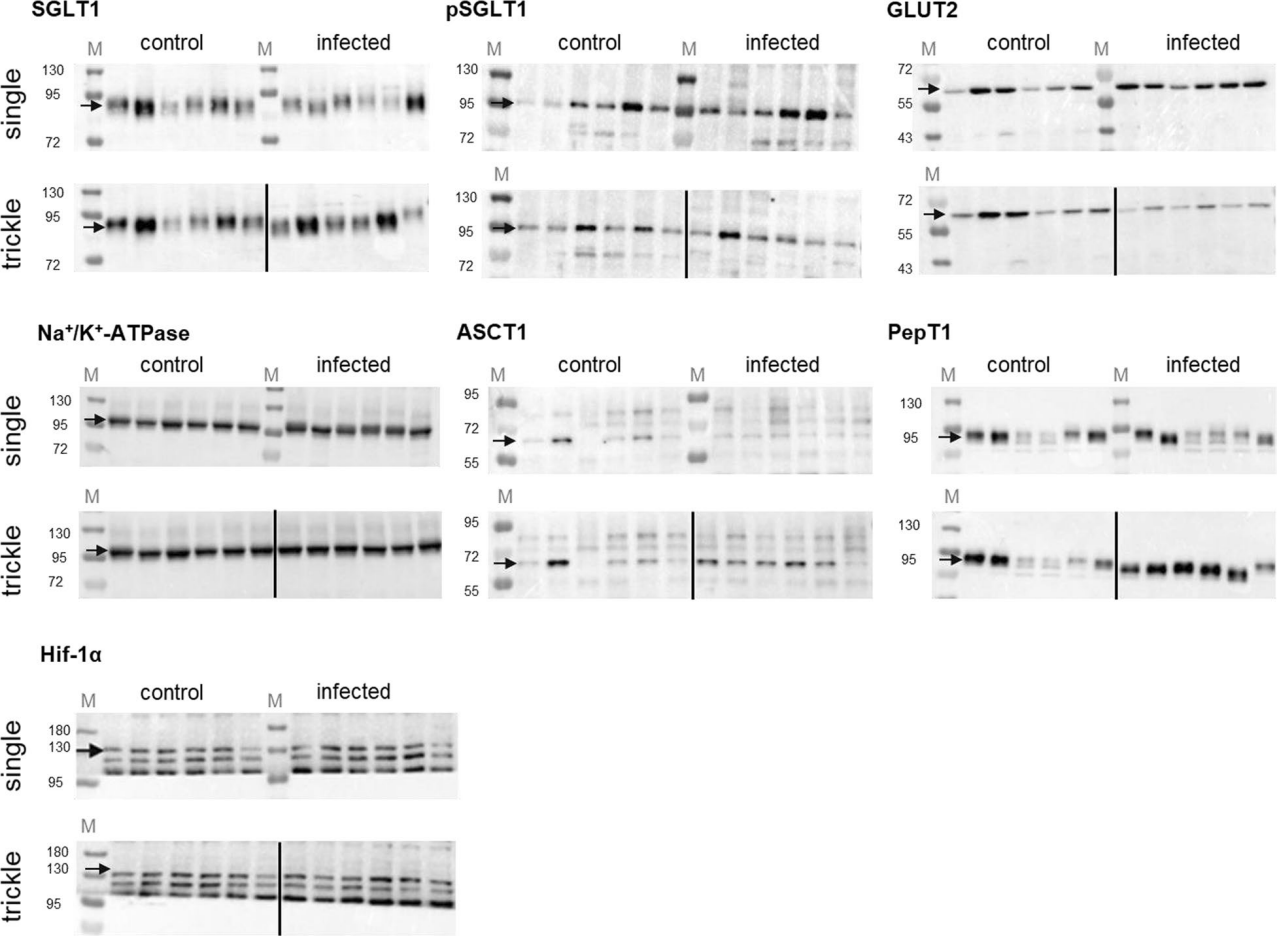
Representative immunoblots conducted on jejunal and ileal mucosa of individual pigs showing bands of SGLT1, pSGLT1, GLUT2, Na^+^/K^+^-ATPase, ASCT1, PepT1 and Hif-1α. Expected molecular masses are indicated with an arrow. Total protein was stained with Indian ink and subtracted from resulting band intensities. The first six lanes to the left of each membrane represent the control animals, while the following six lanes represent the single-infected (top) or the trickle-infected (bottom) animals. M: marker (26616 PageRuler™, Thermo Fisher Scientific, Waltham, USA)

Representative western blots are shown in Fig. 7. At 21 dpi, no significant changes regarding pSGLT1 or SGLT1 expression occurred (Table 7). The trickle-infected group showed a significant increase in SGLT1 expression in the jejunum at 35 dpi (Fig. 2b; Table 8). At 49 dpi, pSGLT1 expression was increased in the ileum of the single-infected group (Fig. 3e; Table 9).

GLUT2 showed a significant increase at 21 dpi in the ileum of the trickle-infected group (Fig. 1e; Table 7) as well as in the jejunum of the single-infected group at 49 dpi (Fig. 3b; Table 9), but a significant decrease in the jejunum at 35 dpi (Fig. 2b; Table 8) and in the ileum at 49 dpi (Fig. 3e; Table 9).

No significant differences in the expression of Na^+^/K^+^-ATPase were observed. The expression of Hif-1α was unaltered at 49 dpi (Fig. 3b, e; Table 9), but significantly increased in the ileum of the single-infected group at 21 dpi (Fig. 1e; Table 7) and both infection groups at 35 dpi (Fig. 2e; Table 8).

#### Expression of peptide (PepT1) and amino acid transporters (ASCT1)

PepT1 expression was significantly decreased in the ileum of the single-infected group at 35 dpi (Fig. 5e; Table 8), but remained unchanged at 21 and 49 dpi, as well as in the trickle-infected group. Regarding ASCT1, no significant changes occurred at 21 dpi or 49 dpi (Figs. 4b, e, 6b, e; Tables 7, 9). At 35 dpi, no obvious upper bands were detected, and expression could not be measured.

### Histomorphometric analysis

Villus length, villus width and crypt depth in jejunal and ileal samples from 49 dpi showed no significant changes in response to infection (Table 10).

**Table 10:** Summary of histomorphometric analysis of jejunal and ileal samples (49 dpi)

	Control	Single infection	Trickle infection	Single infection vs control	Trickle infection vs control
	Mean ± SEM	Mean ± SEM	Mean ± SEM	Mann–Whitney *U*-test	Mann–Whitney *U*-test
49 dpi jejunum					
Total length [µm]	760.3 ± 54.80	796.7 ± 52.34	911.0 ± 60.00	*U* = 14, *P* = 0.589	*U* = 7, *P* = 0.093
Villus length [µm]	475.1 ± 34.10	449.5 ± 47.48	573.2 ± 46.73	*U* = 14, *P* = 0.589	*U* = 8, *P* = 0.132
Crypt depth [µm]	285.2 ± 24.29	347.2 ± 25.64	337.8 ± 31.78	*U* = 12, *P* = 0.394	*U* = 11, *P* = 0.310
Villus width [µm]	140.0 ± 4.61	133.9 ± 2.52	136.8 ± 3.02	*U* = 13, *P* = 0.485	*U* = 16, *P* = 0.818
49 dpi ileum					
Total length [µm]	647.6 ± 28.53	624.2 ± 44.34	662.1 ± 46.06	*U* = 14, *P* = 0.589	*U* = 18, *P* > 0.999
Villus length [µm]	375.9 ± 24.41	330.3 ± 47.59	375.3 ± 20.93	*U* = 10, *P* = 0.240	*U* = 17, *P* = 0.937
Crypt depth [µm]	271.6 ± 21.10	293.9 ± 19.29	286.9 ± 27.41	*U* = 13, *P* = 0.485	*U* = 16, *P* = 0.818
Villus width [µm]	140.0 ± 3.02	135.1 ± 2.06	145.6 ± 5.61	*U* = 13, *P* = 0.485	*U* = 12, *P* = 0.394

## Discussion

This project aimed at characterizing the intestinal electrophysiological response to glucose, peptides and alanine as well as the transcription and expression of cytokines and nutrient transporters following experimental *A. suum* infection. To gain a comprehensive picture, different small intestinal segments and different time points pi to cover all gut-associated parasite stages, as well as a single infection and a more natural trickle infection were considered. The presence of milk spots indicated successful infection of pigs, while the serological response was somewhat variable, with most infected pigs seroconverting between 7 and 21 dpi, but some becoming seropositive as late as 42 or 49 dpi. As the ELISA test utilized was designed for use in humans, further validation and cut-off adjustment may be necessary regarding *A. suum* infection in pigs.

The histomorphometric measurements revealed no differences in villus length or crypt depth at 49 dpi. Thus, changes in mucosal surface in response to the infection are unlikely.

Regarding glucose transport, no significant effect of *A. suum* infection was observed in the present study. This is in contrast to previous studies in *A. galli*-infected chickens as well as *A. suum*-infected pigs, which showed a decrease in the electrogenic response to glucose as compared to nematode-free animals [14, 15].

Nevertheless, western blot analysis showed an upregulation of relevant transporters, e.g. GLUT2 in the ileum of the trickle-infected group at 21 dpi and in the jejunum of the single-infected group at 49 dpi. Furthermore, *SGLT1* transcription was increased in the jejunum of both infection groups, while transcription of *GLUT1* and *Hif-1α* was upregulated in the trickle-infected group at 21 dpi. An increase in both *SGLT1* and *GLUT1* transcription was also observed in the ileum of the trickle-infected group at 21 dpi. The increase in both *SGLT1* and *GLUT1* transcription seemingly contradicts previous findings, because GLUT1 is supposed to be upregulated in order to compensate for the downregulation of SGLT1 [33]. It has to be considered, however, that the increased *SGLT1* transcription observed in the current study does not necessarily indicate increased expression and therefore altered glucose transport. SGLT1 phosphorylation was also examined, as pSGLT1 is a more active transporter [21]. However, the ratio of SGLT1 and pSGLT1 remained unaffected, corroborating the unaltered Δ*I*_sc_.

Furthermore, distinct differences were observed between the jejunum and ileum. In the jejunum, GLUT2 showed significantly increased expression in the single-infected group and significantly increased transcription in the trickle-infected group at 49 dpi, while a significant decrease in GLUT2 expression was noted in the ileum of the trickle-infected group. These differences might be related to the different functions of the intestinal segments. While the proximal parts of the small intestines are exposed to higher glucose levels, the distal parts receive chyme with lower glucose content [21]. Therefore, the reaction and distribution of glucose transporters might vary depending on the segment examined. Earlier studies reported stronger electrogenic responses in the ileum than in the jejunum of pigs [34, 35]. Most previous studies on nematode-related alterations of glucose transport, however, were performed only with jejunal tissue [14, 17, 33], precluding any comparison with previous studies regarding the ileum.

Because (p)SGLT1 is coupled with a sodium gradient, sodium needs to be exported to maintain the driving force for the glucose transporters. The expression of Na^+^/K^+^-ATPase was analysed, but no significant changes were observed. Therefore, a changed expression of Na^+^/K^+^-ATPase seems unlikely to create an altered gradient. Nevertheless, a change in activity, e.g. by PKA phosphorylation, cannot be excluded [36].

Earlier studies on the effect of IL-4 and IL-13 on sodium-coupled glucose absorption showed decreased glucose transport in wild-type but not in STAT6-deficient mice [17]. Therefore, elevated production of the STAT6 activators IL-4 and IL-13 might affect glucose transport. Interestingly, during the early phase of infection (21 dpi), *STAT6* transcription was significantly upregulated in both segments in the trickle-infected group, supporting this hypothesis. Nevertheless, a significant decrease in *IL-13* transcription in the jejunum and ileum of trickle-infected pigs occurred at 49 dpi, whereas *IL-4* was significantly increased in the ileum, complicating the interpretation of the findings. In a study on *A. galli*-infected chickens [15], an increase in *IL-4 *and *IL-13* transcription was observed 2 to 3 weeks pi. However, *A. galli* does not perform a body migration but develops in the intestine only. Considering the body migration of *A. suum* and its arrival in the small intestine at ~8 dpi, typical reactions may occur around 35 dpi, conceivably explaining the significant increase in IL-4 observed at this time point in the current study.

A reduction of electrogenic peptide transport was observed at 49 dpi, underlining the hypothesis of nutrient malabsorption due to helminth infection [14, 15]. Both infected groups showed a significant reduction in the ileum, and the trickle-infected group additionally in the jejunum. Since PepT1 expression in the ileum remained unchanged at 49 dpi in both infection groups, the question arises as to whether phosphorylation of NHE3 by PKA might impair the maintenance of the H^+^ gradient and thus peptide transport [20]. This could lead to a reduced function of PepT1, without changing mRNA transcription or protein expression. However, PKA activity, as measured by western blot analysis of phosphorylated PKA substrates, was not clearly changed after *A. suum* infection, and the phosphorylation of NHE at the respective phosphorylation sites was mostly unaffected and not correlated with functional changes (data not shown).

Regarding peptide transport, both segments of trickle-infected pigs showed a significant increase in *PepT1* transcription at 21 dpi. It is possible that the nematode has a high demand for peptides at this stage of development, leading to more intense host–parasite competition for these nutrients. Promoting PepT1 and therefore peptide uptake could be a counterregulatory mechanism of the host to compensate for the parasitic infection. On the level of protein expression, however, no significant changes in PepT1 were observed, with the exception of a reduction in the ileum of the single-infected group at 35 dpi.

Similar to the alterations observed regarding peptide transport, alanine transport was also significantly decreased at 49 dpi in the trickle-infected group. However, the decrease cannot be explained by the transcriptional and expression data, since these revealed no significant changes. It could be speculated that the parasites affect the epithelial tissue unevenly. However, since *A. suum* resides in the gut lumen without being locally attached, it seems reasonable that the intestinal tissue is evenly exposed to the parasites’ excretory-secretory antigens. Based on the induced hypercontractility [37] related to the nematodes’ presence, this seems probable. Further immunoregulatory processes may play an important role. The Th2-associated release of IL-4 and IL-13 is one example among other possible mechanisms [17, 38, 39]. Hence, monitoring of respective immune parameters, for example mast cells [33], might be a valuable approach for future studies. It is known that parasitic antigens cause degranulation of IgE-sensitized mast cells [18]. The released cytokine LTD4 supposedly leads to a phosphorylation of ASCT1 mediated by PKA and/or PKC-α, which results in the downregulation of ASCT1 activity based on decreased affinity [19]. This may be an explanation for the observed reduced Na-alanine cotransport.

Overall, the trickle infection had a greater impact on the results, highlighting that single, high-dose infections may not be ideal for studying host–parasite interactions. This is in accord with previous reports that trickle infections resulted in more successfully manifested *A. suum* infections than single infections, which induce a strong expulsive reaction of the host [40, 41]. However, as discussed below, it was not possible to quantify worm burden in the current study to confirm this hypothesis.

Furthermore, more differences were observed towards the end of the study, indicating that the parasites’ impact may increase with the length of infection. Thus, the pigs may have been slaughtered too early for differences in weight gain to become apparent, namely before or just at the beginning of patency, when most worms were still immature. Adult worms may have a different or stronger impact on the transport processes, compared with larval or subadult stages. In line with this theory, the parasite’s excretory-secretory antigens are heterogeneous in their molecular weight specific to the developmental stage of the parasite [42].

Moreover, it has to be considered that the results in general, but especially regarding Δ*I*_sc_, showed a high variance. One shortcoming of the study is that individual parasite burdens could not be quantitatively assessed, due to the need to rapidly transfer the mucosa to Ussing chambers. Therefore, parasites were not counted during the sampling procedure. Immunologically mediated parasite expulsion between days 14 and 21 pi leads to considerable variation between individuals, and some animals may even completely eliminate the infection [4, 43, 44], which may have caused the large variance in the results. Therefore, a higher number of experimental animals and thus larger sample size might allow for more resilient conclusions in future studies, also with regard to differing parasite burdens between individual animals.

## Conclusions

Considering electrophysiological, transcriptional and expression data as well as weight gain and histomorphometric analysis, the infected pigs did not experience the comprehensive nutrient restriction that was expected based on previous studies [14, 15]. Surprisingly, the weight gain was even significantly increased at 49 dpi in the trickle-infected group. Therefore, the common clinical finding of reduced weight gain in *A. suum* field studies cannot be fully explained by alterations in nutrient transport. Nevertheless, significant differences between the experimental groups were observed, especially regarding electrogenic peptide and alanine transport. This included a tendency towards an upregulation of the PepT1-mediated transport of gly-gln and PepT1 expression at 21 dpi, followed by downregulation at later points of infection. These changes occurred in the jejunum and ileum, whereas no changes were measured in the duodenum. Moreover, the observed functional alterations were not fully explained by transcriptional or expression changes. Thus, further studies are necessary to fully elucidate the mechanisms of nutrient restriction during *Ascaris* infections.

## Data Availability

All data that was generated and analysed in this study are available from the corresponding author on reasonable request.

## Abbreviations

ASCT1: Neutral amino acid transporter A
BSA: Bovine serum albumin
cDNA: Complementary deoxyribonucleic acid
Cl^−^: Chloride
dpi: Days post-infection
DTT: Dithiothreitol
GLUT: Glucose transporter 1
gly-gln: Glycyl-l-glutamine
IL: Interleukin
Isc: Short-circuit current
Jms: Mucosal-to-serosal flux rate
Jnet: Net flux rate
Jsm: Serosal-to-mucosal flux rate
MGB: Minor groove binder
MP: Milk powder
mRNA: Messenger ribonucleic acid
Na^+^/K^+^-ATPase: Sodium–potassium adenosine triphosphatase
NCBI: National Center for Biotechnology Information
qPCR: Quantitative polymerase chain reaction
PBS: Phosphate-buffered saline
PepT1: Peptide transporter 1
PKA: Protein kinase A
PKC: Protein kinase C
PPIA: Peptidylprolyl isomerase A
pSGLT1: Phosphorylated sodium-dependent glucose cotransporter 1
RNAse: Ribonuclease
RT: Room temperature
SDS: Sodium dodecyl sulfate
SGLT1: Sodium-dependent glucose cotransporter 1
STAT6: Signal transducer and activator of transcription 6
TBP: TATA-binding protein
TBS: Tris-buffered saline
Th2: T helper cell 2
TRIS: Tris-hydroxymethyl aminomethane
